# High-Throughput SARS-CoV-2 Antiviral Testing Method Using the Celigo Image Cytometer

**DOI:** 10.1007/s10895-023-03289-x

**Published:** 2023-06-13

**Authors:** Laura A. St Clair, Leo Li-Ying Chan, Adam Boretsky, Bo Lin, Michael Spedding, Rushika Perera

**Affiliations:** 1 Center for Vector-borne Infectious Diseases, Department of Microbiology, Immunology, and Pathology, Colorado State University, CO 80523 Fort Collins, USA; 2 Center for Metabolism of Infectious Diseases (C4MInD), Colorado State University, 3185 Rampart Rd, Fort Collins, CO 80523 USA; 3 Department of Advanced Technology R&D, Revvity, 360 Merrimack St. Suite 200, Lawrence, MA 01843 USA; 4 Spedding Research Solutions SAS, Le Vesinet, France

**Keywords:** SARS-CoV-2, COVID-19, Antiviral, Drug repurposing, Image cytometry, Celigo

## Abstract

The COVID-19 pandemic has created a worldwide public health crisis that has since resulted in 6.8 million reported deaths. The pandemic prompted the immediate response of researchers around the world to engage in rapid vaccine development, surveillance programs, and antiviral testing, which resulted in the delivery of multiple vaccines and repurposed antiviral drug candidates. However, the emergence of new highly transmissible SARS-CoV-2 variants has renewed the desire for discovering new antiviral drug candidates with high efficacy against the emerging variants of concern. Traditional antiviral testing methods employ the plaque-reduction neutralization tests (PRNTs), plaque assays, or RT-PCR analysis, but each assay can be tedious and time-consuming, requiring 2–3 days to complete the initial antiviral assay in biologically relevant cells, and then 3–4 days to visualize and count plaques in Vero cells, or to complete cell extractions and PCR analysis. In recent years, plate-based image cytometers have demonstrated high-throughput vaccine screening methods, which can be adopted for screening potential antiviral drug candidates. In this work, we developed a high-throughput antiviral testing method employing the Celigo Image Cytometer to investigate the efficacy of antiviral drug candidates on SARS-CoV-2 infectivity using a fluorescent reporter virus and their safety by measuring the cytotoxicity effects on the healthy host cell line using fluorescent viability stains. Compared to traditional methods, the assays defined here eliminated on average 3–4 days from our standard processing time for antiviral testing. Moreover, we were able to utilize human cell lines directly that are not typically amenable to PRNT or plaque assays. The Celigo Image Cytometer can provide an efficient and robust method to rapidly identify potential antiviral drugs to effectively combat the rapidly spreading SARS-CoV-2 virus and its variants during the pandemic.

**Supplementary Information:**

The online version contains supplementary material available at 10.1007/s10895-023-03289-x.

## Introduction

Since January of 2020, the COVID-19 pandemic has created a major public health crisis across the globe. Since the start of the pandemic, approximately 766 million cases and 6.9 million deaths have been reported worldwide [1]. In response, researchers around the world have engaged in rapid vaccine development, surveillance programs, and antiviral testing. These combined efforts within the community led to the development and delivery of multiple vaccine candidates, where an estimated 13.3 billion doses of vaccines have been administered worldwide [1]. To date, there have been numerous drugs tested and repurposed as antivirals that have either received approval or emergency use authorization such as remdesivir, molnupiravir, nirmatrelvir/ritonavir (Paxlovid™), while testing and clinical trials are ongoing for many others [2–5]. However, each of these drugs has its limitations for use, and are not yet available to many low-income communities throughout the world. Additionally, with the emergence of new highly transmissible variants that are showing an increased ability to circumvent the immunity conferred by vaccination, like the Omicron variants that emerged beginning in November 2021, there has been a renewed interest in the discovery of novel antiviral candidates effective against emerging variants of concern.

Traditional antiviral or vaccine testing methods utilize plaque-reduction neutralization tests (PRNTs), plaque assays or RT-PCR analysis to investigate the effects of antiviral drugs and vaccines to neutralize viral infections. However, each assay is time-consuming, requiring days to either form visually clear plaques or to prepare cell extractions for PCR analysis. These assays are also low-throughput and can have high operator-to-operator variation. Furthermore, digital images of the viral reduction are not typically recorded [6]. These facts underpin a critical need to develop high-throughput testing methods for new antiviral drugs and vaccine candidates to improve the efficiency of research and development.

Plate-based image cytometry systems have previously demonstrated high-throughput screening for potential vaccine candidates [6–9]. The Celigo Image Cytometer (Revvity, formerly known as Nexcelom Bioscience, Lawrence, MA) has been employed for high-speed and high-throughput counting of viral plaques, foci, and individual virus-infected cells in 96-well microplates using bright field or fluorescent imaging. Image cytometric analysis can significantly reduce the plaque counting time and minimize operator-to-operator variation, which can improve the efficiency of identifying new or novel therapeutic candidates for viral diseases [10–12]. Current publications have shown the improvement in assay time and precision for fluorescent detection of plaques, foci, or individual infected cells via immunostaining or fluorescent protein reporter for COVID-19 vaccine development [13, 14]. In this work, we developed a high-throughput antiviral testing method employing the Celigo Image Cytometer to investigate the effects of antiviral drugs candidates on infection rates using a SARS-CoV-2 reporter virus that stably expresses a fluorescent mNeonGreen reporter protein as well as their cytotoxicity effects on the healthy host cell line using fluorescent viability stains. The host cell line, multiplicity of infection (MOI), and cell seeding density were first selected for antiviral testing during method development. The optimized image cytometry method was then used to demonstrate antiviral testing of four drug compounds repurposed for potential COVID-19 treatment at different concentrations. The high-throughput antiviral testing method using Celigo Image Cytometer can provide a more efficient platform to rapidly identify potential antiviral drugs, which is highly critical during a global pandemic to combat the rapidly spreading SARS-CoV-2 virus and its variants.

## Materials and Methods

### Cell Lines and Viruses Used

The cell lines used to develop the image cytometry method for SARS-CoV-2 antiviral testing have been published previously: Human lung adenocarcinoma cells stably transfected to express ACE2 (ACE2-A549s) [15], Human lung adenocarcinoma (Calu3) cells (ATCC HTB-55), two human pharyngeal carcinoma (Fadu, Detroit 562) cell lines (ATCC HTB-43 and ATCC CCL-138), human hepatoma (Huh7) cells (unknown sex, a gift from Dr. Charles Rice) [16], and African green monkey kidney epithelial (Vero E6) cells (ATCC CRL-1586).

ACE2-A549s, Huh7, and Vero E6 cells were maintained in Dulbecco’s Modified Eagle Medium (DMEM) (Gibco/Life Technologies, Carlsbad, CA) and supplemented with 10% heat-inactivated fetal bovine serum (FBS) (Atlas Biologicals, Fort Collins, CO). Calu3 cells were maintained in DMEM and supplemented with 15% non-heat inactivated FBS. Fadu and Detriot 562 cells were maintained in Minimum Essential Media (MEM) (Gibco/Life Technologies) supplemented with 10% non-heat inactivated FBS. All media was also supplemented with 2 mM nonessential amino acids (HyClone, Logan, UT), 2 mM L-glutamine (HyClone), and 25 mM HEPES buffer. All cell lines were maintained at 37 °C with 5% CO_2_. All cell culture and cell seeding were performed in our BSL-2 facility while all experiments involving SARS-CoV-2 infection or use of the Celigo Imaging Cytometer occurred after transfer of seeded plates to our BSL-3 facility.

The mNeonGreen SARS-CoV-2 virus strain was provided by the World Reference Center for Emerging Viruses and Arboviruses at the University of Texas Medical Branch, Galveston, TX [17]. This infectious clone was constructed by Xie et al. based on the virus strain (2019-nCOV/USA_WA01/2020) isolated from the first reported SARS-CoV-2 case in the US [18–20]. Viral stocks were amplified in Vero E6 cells to passage 1 (P1) with a titer of 9.67 × 10^5^ PFU/mL and were stored at -80 °C. Viral infections were carried out at specific multiplicity of infection (MOI) and incubation time for assay development. During all infections, cells were overlaid with either MEM or DMEM supplemented with 2% heat-inactivated FBS, 2 mM non-essential amino acids, 2 mM L-glutamine, and 25 mM HEPES buffer.

### Celigo Image Cytometer and Image Analysis Software

The Celigo Image Cytometer has been described previously, which utilizes one bright field (BF) and four fluorescence (FL) imaging channels: Blue (Ex/Em: 377/470 nm), Green (Ex/Em: 483/536 nm), Red (Ex/Em: 531/629 nm), and Far Red (Ex/Em: 628/688 nm) in combination with high-power light-emitting diodes [21–24]. The image cytometry system acquires whole well (96-well plate) images by utilizing a proprietary mirror system that rapidly captures 16 images in one well, where the software algorithm automatically stitches them together for visualization. The entire imaging system provides an optical resolution of ~ 1 μm^2^/pixel.

The Celigo software application “Confluence 1” was used to measure the host cell confluence percentages using the acquired bright field images. The preset ANALYZE parameters were optimized to automatically measure confluence percentages. The confluence percentages were calculated as the ratio of cell surface area coverage divided by total surface area in the well measured directly from the image cytometer. The results were used to optimize Calu3 seeding density.

The Celigo software application “Target 1 + 2 + Mask” was used to identify the total number of Hoechst-positive host cells (Ex/Em: 352/461 nm) in the Blue channel and the number of mNeonGreen fluorescent infected cells (Ex/Em: 506/517 nm) in the Green channel. The Celigo instrument was set up to acquire images in the Target 1 (BF), Target 2 (Green), and Mask (Blue) channels, where the exposure times for mNeonGreen and Hoechst were 60,000 and 145,000 µs, respectively. Image-based autofocus was used to focus in the BF channel, and the focus offsets were applied for the BF (-2 µm), Green (0 µm) and Blue (-15 µm) channels. The preset ANALYZE parameters were optimized to identify the Hoechst-positive host cells above an intensity threshold of 2, then the gating feature was used to determine the mNeonGreen-positive infected cell number.

The Celigo software application “Target 1 + 2 + 3 + 4” was used to identify the total number of Hoechst-positive cells in the Blue channel, the number of live/metabolically active Calcein AM-positive cells (Ex/Em: 488/520 nm) in the Green channel, and dead propidium iodide-positive cells (PI, Ex/Em: 496/636 nm) in the Red channel. The Celigo instrument was set up to acquire images in the Target 1 (BF), Target 2 (Blue), Target 3 (Green), and Target 4 (Red) channels, where the exposure times for Hoechst, Calcein AM, and PI were 23,000, 10,000, and 10,000 µs, respectively. Image-based autofocus was used to focus in the BF channel, and the focus offsets were applied for the BF (-8 µm), Blue (-13 µm), Green (+ 7 µm) and Red (0 µm) channels. The preset ANALYZE parameters were optimized to identify the fluorescent positive cells above an intensity threshold of 4.

### Host Cell Line and MOI Selection

Multiple host cell lines were infected with different MOIs of the mNeonGreen SARS-CoV-2 virus to select the most appropriate cell type for developing the antiviral testing method. First, the ACE2-A549, Calu3, Huh7, Fadu, Detroit 562, and Vero E6 cell types were seeded in a 96-well plate (Greiner 655,180) at 20,000 cells/well and allowed to adhere overnight under BSL-2 conditions. After 24 h post-seeding, the ACE2-A549, Calu3, and Huh7 were transferred to our BSL-3 facilities and were either uninfected or infected with MOIs of 0.01, 0.1, and 1. The Fadu and Detroit 562 were either uninfected or infected with MOIs of 0.001, 0.01, 0.1, and 1. All SARS-CoV-2 infections and Celigo assays were performed within BSL-3 containment. After 72 h post-infection (hpi), the cells were stained with ViaStain™ Hoechst 33,342 (Revvity, Lawrence, MA) following manufacturer’s instructions. These protocols can be used with or without fixing cells depending on user preference, but cells were not fixed in these experiments outlined in this paper so that live-cell imaging could be acquired. Subsequently, the wells were scanned using the image cytometer to determine the total cell count and infected cell count for selecting the most optimal cell type. The infected cell count results were normalized to the average total and infected cell count for the control (uninfected). Uninfected cells were utilized as the control in order to allow for correction of background green fluorescence from cells. Each cell line and MOI was tested at n = 3, and the degree of viral infection was calculated with the equation ${\mathrm{Log}}_{10}\left(mNeonGreen\, count\right)= {\mathrm{Log}}_{10}\left(\frac{\frac{{Hoechst}_{Sample}^{+}}{AVE \,{Hoechst}_{Control}^{+} }\times {mNeonGreen}_{Sample}^{+}}{AVE\, {mNeonGreen}_{Control}^{+}}\right)$, where the counted results were normalized to the total cell count (AVE Hoechst^+^_Control_) and uninfected control (AVE mNeonGreen^+^_Control_).

### Cell Seeding Density Selection

A cell proliferation experiment was conducted to optimize the seeding density for the antiviral testing method using Calu3 that was selected from the experiment described previously. The Calu3 cells were seeded at densities of 1 × 10^5^, 8 × 10^4^, 6 × 10^4^, 4 × 10^4^, 2 × 10^4^, and 1 × 10^4^ cells/well in a 96-well plate (n = 3). The cells were allowed to incubate for 96 h post-seeding, where the plate was scanned using the image cytometer at 24, 72, and 96 h. The confluence percentages were measured at each time point to select a seeding density that can generate 50 – 70% confluence.

### High-Throughput Image-Based Antiviral Testing method

To demonstrate the optimized antiviral testing method, an experiment was performed to measure the antiviral effects of four drugs on SARS-CoV-2-infected Calu3 cells. Remdesivir (#30354, Cayman Chemical, Ann Arbor, MI), Ribavirin (#R9644, Sigma-Aldrich, St. Louis, MO), Ambroxol hydrochloride (#A0363700, Sigma-Aldrich, St. Louis, MO), and GENZ-123346 (#28500, Cayman Chemical, Ann Arbor, MI) were selected for testing the image-based method. Remdesivir, Ambroxol HCl, and GENZ-123346 were solubilized in dimethyl sulfoxide (DMSO), while Ribavirin was solubilized in dimethylformamide (DMF) according to manufacturer’s guidance on compound solubility. Remdesivir and Ribavirin were selected for the assay as positive controls since they were already shown to be relatively effective at inhibiting SARS-CoV-2 in vitro [25, 26]. Ambroxol HCl and GENZ-123346 are both modulators of glucosylceramide, a lipid metabolite that may be vital for SARS-CoV-2 replication [27]. Ambroxol HCl is a molecular chaperone of β-glucocerebrosidase 1, and inhibitor of β-glucocerebrosidase 2, and can modulate glycosphingolipid metabolism in pathophysiological situations by these mechanisms [28]. GENZ-123346 is an inhibitor of glucosylceramide synthase. These compounds were chosen as part of an ongoing study in our laboratory investigating the role of sphingolipid metabolism in SARS-CoV-2 infections.

The Calu3 cells were first seeded at a density of 2 × 10^5^ cells/well in 96-well plates and incubated overnight. Approximately 1 – 3 h prior to infection, the growth media was removed from cells, and subsequently overlaid with DMEM supplemented with 2% heat-inactivated FBS, 2 mM NEAA, 2 mM L-glutamine, 25 mM HEPES buffer, containing each compound at different concentrations: Remdesivir (10, 5, 1, 0.5 μM), Ribavirin (400, 200, 100, 50 μM), Ambroxol HCl (100, 50, 25, 12.5 μM), and GENZ-123346 (50, 25, 12.5, 6.25 μM). The concentrations selected were based on published IC_50_ values for Remdesivir and Ribavirin [25] and previous optimization in our studies for Ambroxol HCl and GENZ-123346. The cells were then infected with the mNeonGreen SARS-CoV-2 virus at MOI of 0.1 for 72 h. During these experiments, the media overlay was not changed following initial addition of the antiviral compounds and viral inoculum, and all calculations were conducted to allow for a final volume of 100 μL total volume per well of media + antiviral and/or vehicle overlay and viral inoculum in order to control for the concentration of each antiviral compound tested. Each drug compound and its corresponding vehicle control (DMSO or DMF) were tested in triplicate (n = 3). It is important to note that the 96-well plates were made in duplicate to investigate the antiviral activity as well as assessing cytotoxicity effects of the compounds on host cells in parallel.

Following the infection experiment, the supernatant from infected Calu3 cells was harvested and stored at -80 °C. The remaining cells were washed with 1X PBS, and then stained with the ViaStain™ Hoechst 33,342 following manufacturer’s instructions. To determine viral replication, the plates were imaged and analyzed using the image cytometer to count the total number of Calu3 cells with Hoechst staining and mNeonGreen SARS-CoV-2 infection. The infected cell count results were normalized to the average total cell count.

In contrast, the cytotoxicity plates were not infected with the mNeonGreen reporter virus, but all other conditions and timeframes were maintained. Following the experiment, the media from uninfected cells was aspirated, cells were washed with 1X PBS, and then were stained with the ViaStain™ Calcein AM/Hoechst/PI Viability Kit following manufacturer’s instructions. The viability kit allowed for determination of the effects of various compounds and their respective concentrations on host cell viability. To determine the cell viability, the plates were imaged and analyzed using the image cytometer to count the total number of cells with Hoechst, the live cells with Calcein AM, and dead cells with PI. The live and dead cell count results were also normalized to the average total cell count.

### Statistical Analysis

The statistical analysis method used is noted in each applicable figure. Determination of statistical significance for antiviral effect, cell viability, and live/dead cell counts was performed using a one-way Analysis of Variance (ANOVA) with Dunnett’s multiple comparisons using version 9.3.1 of Prism software (GraphPad Software, La Jolla, California, USA).

## Results

### Selection of the Optimal Host Cell Line and MOI

**Fig. 1:**
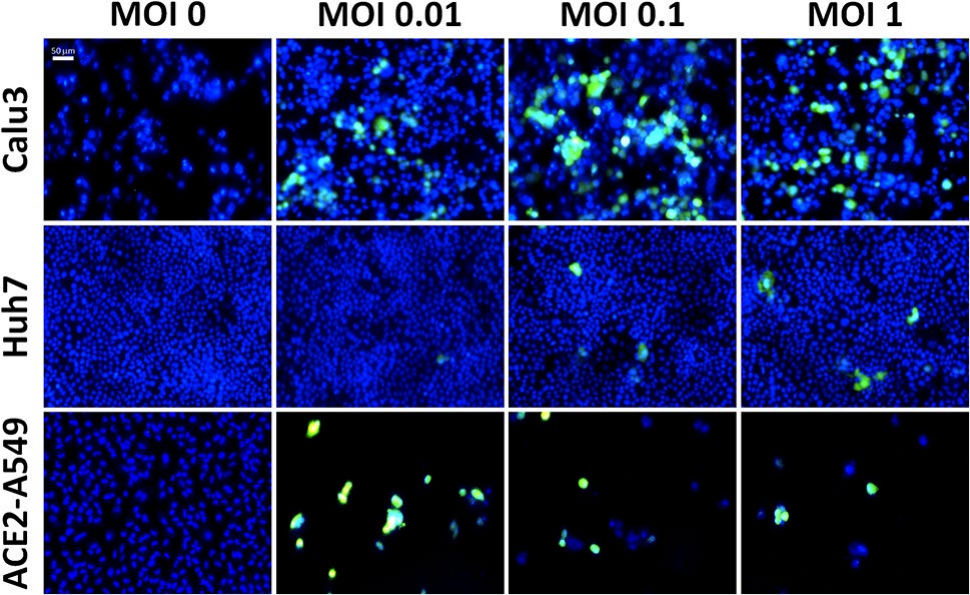
Fluorescent overlay images of Hoechst and mNeonGreen SARS-CoV-2-infected Calu3, Huh7, and ACE2-A529 at MOIs 0.01, 0.1, and 1 at 72 hpi. Visually, Calu3 and ACE2-A549 cells both showed permissibility to the SARS-CoV-2 virus, but only the ACE2-A549 showed significant cell loss

To develop a high-throughput SARS-CoV-2 antiviral testing method, we first sought to determine an appropriate host cell line and the respective MOI. The purpose was to identify the potential cell types that were permissive to infection with the mNeonGreen SARS-CoV-2 reporter virus and provide robust and biologically relevant results for the antiviral testing method. In this initial experiment, we compared the infection of Calu3, Huh7, ACE2-A549, Detroit 562, and Fadu cells at various MOIs. The fluorescent overlay images of Hoechst and mNeonGreen are displayed in Fig. 1, which visually showed higher infection for Calu3 in comparison to Huh7. The ACE2-A549 also showed high infection, however, a significant cell loss was observed. It is important to note that these are representative fluorescent overlay images from the image cytometer.

**Fig. 2:**
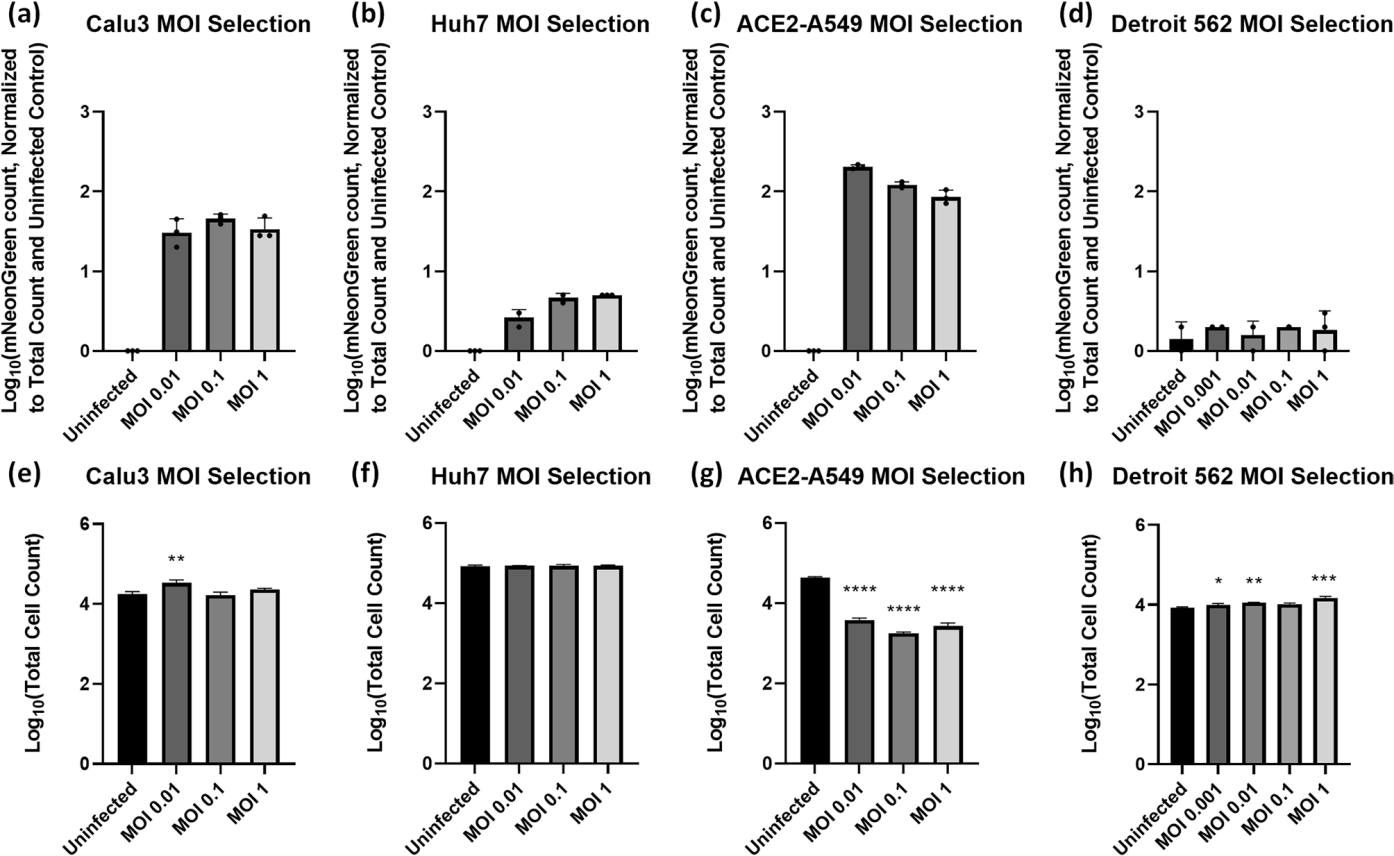
Cell counting results measured by the image cytometer. **a**–**d** The mNeonGreen positive cell counting results normalized to the total cell count (**e**–**h**) measured by Hoechst staining. The Calu3 and ACE2-A549 cells showed significant increase to the mNeonGreen positive cell count, while Huh7 and Detroit 562 did not. The ACE2-A549 also showed a significant reduction in the total cell count. E–H: One way ANOVA with Dunnett’s multiple comparisons test: * = p ≤ 0.05, ** = p ≤ 0.01, *** = p ≤ 0.001, **** = p ≤ 0.0001)

The normalized mNeonGreen cell count results are shown in Fig. 2a–d, which indicated that ACE2-A549, Huh7, and Calu3 were permissive to SARS-CoV-2 at low MOI and were viable candidate host cell lines. The Detroit 562 and Fadu cell lines did not show any significant mNeonGreen fluorescence and count, indicating that they were not permissive to SARS-CoV-2 infection (Supplementary Fig. 1). In addition, we observed significant cytopathic effect in SARS-CoV-2-infected ACE2-A549 cells causing cell loss that was not observed in other human cell lines (Fig. 2e–f), which suggested that overexpression of ACE2 in these cells may increase SARS-CoV-2 replication kinetics. The Calu3 cells represent a more biologically relevant cell line (compared to Huh7 cells) and are naturally permissive to infection [29], and thus were selected as the most appropriate cell line for the antiviral testing method. The MOI of 0.1 for Calu3 was also selected based on its lower standard deviation.

### Optimization of Cell Seeding Density

**Fig. 3:**
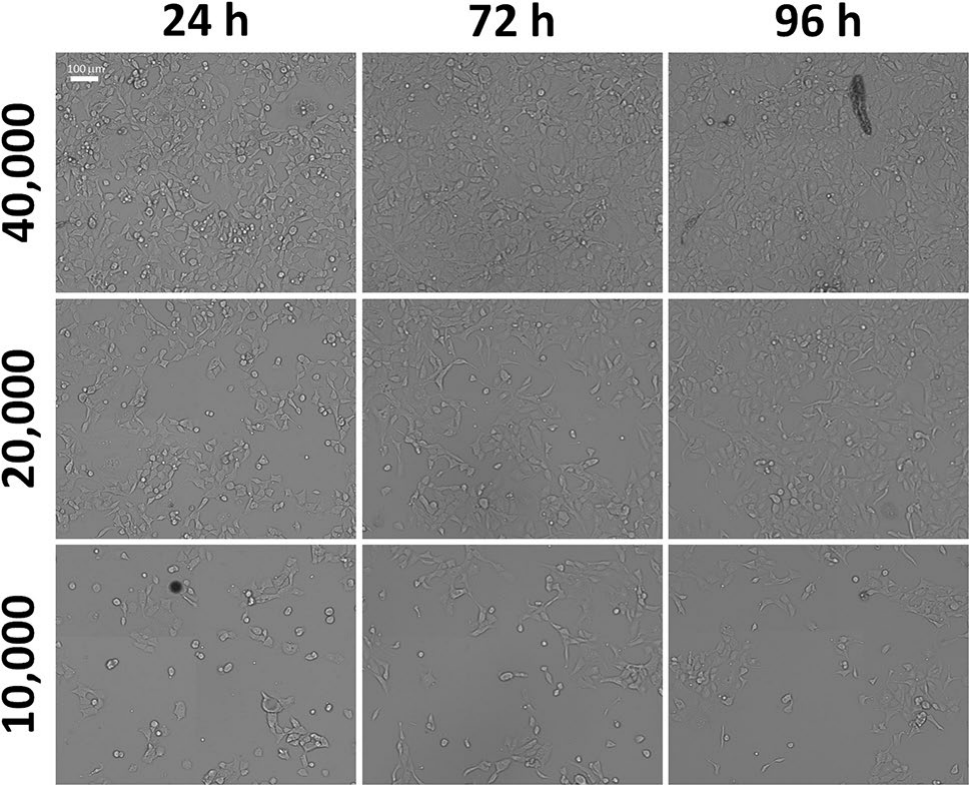
The bright field images of Calu3 confluency at seeding densities 1 × 10^4^, 2 × 10^4^, and 4 × 10^4^ cells/well from 24 to 96 h. Visually, 2 × 10^4^ cells/well is the most appropriate density

**Fig. 4:**
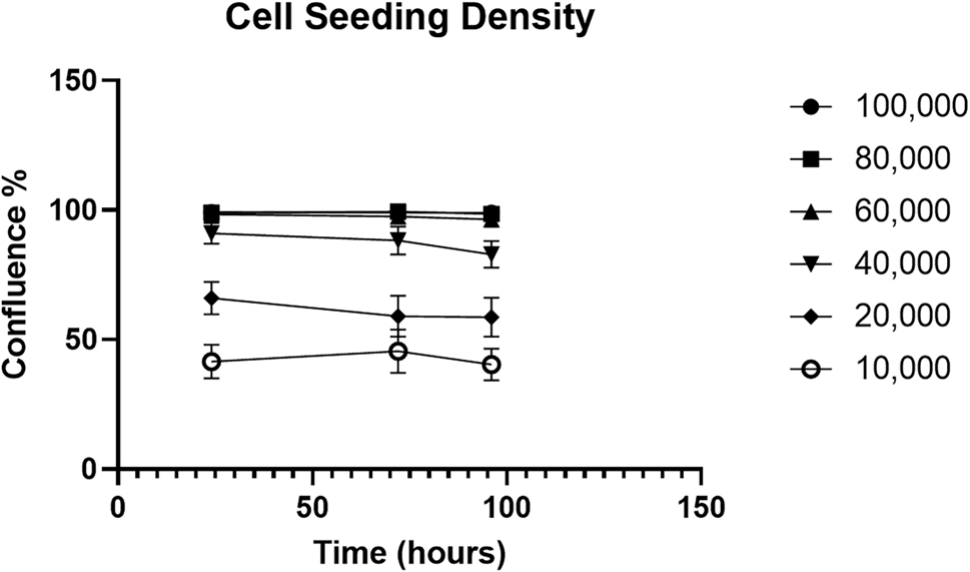
Time-course confluence percentages of Calu3 at seeding densities from 1 × 10^4^ to 1 × 10^5^ cells/well. The 2 × 10^4^ cells/well showed a consistent 60 – 70% confluence over time, while densities at 4 × 10^4^ or above showed closer to 100% confluence

To further optimize the image-based antiviral testing method, we performed a cell seeding density experiment to determine the optimal cell seeding per well. It was previously demonstrated that SARS-CoV-2 has a peak viral replication cycle at approximately 36—72 h post-infection (hpi) [29]. Therefore, the seeding density should allow for adequate detection of the antiviral’s efficacy, visualization of the viral spread, and should not result in overgrowth/cell stress by 72 hpi. The cell proliferation bright field images are shown in Fig. 3 and Supplementary Fig. 2 for 1 × 10^5^, 8 × 10^4^, 6 × 10^4^, 4 × 10^4^, 2 × 10^4^, and 1 × 10^4^ cells/well. For seeding densities above 4 × 10^4^ cells/well at 96 h post seeding (representing 72 h post infection), the confluence percentages were 85 – 100%, which may stress the host cells (Fig. 4). The seeding density of 2 × 10^4^ cells/well provided a clear visualization of host cells in the well and did not show overgrowth over the 96 h.

### Demonstration of the High-Throughput Antiviral Testing Method

**Fig. 5:**
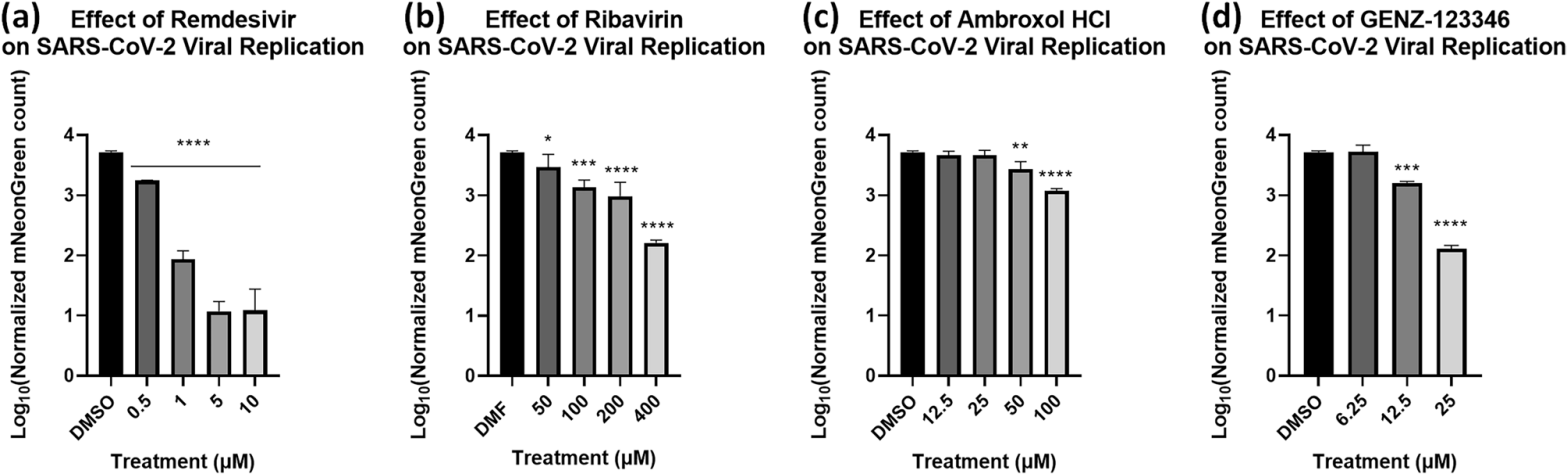
Concentration-dependent effects of drug compounds on SARS-CoV-2 viral replication for **a** Remdesivir, **b** Ribavirin, **c** Ambroxol HCl, and **d** GENZ-123346. All the tested drug compounds showed reduction in the mNeonGreen positive cells, with the following ranking: Remdesivir > Ribavirin ≅ GENZ-123346 > Ambroxol HCl (One way ANOVA with Dunnett’s multiple comparisons test: * = p ≤ 0.05, ** = p ≤ 0.01, *** = p ≤ 0.001, **** = p ≤ 0.0001)

The selected host cell line (Calu3), MOI (0.1), and seeding density (2 × 10^4^ cells/well) were employed to develop the antiviral testing method. To demonstrate the image-based antiviral testing method, we investigated the effects of four drug compounds that were repurposed for treatment of SARS-CoV-2 infection. Each of the drug compounds (Remdesivir, Ribavirin, Ambroxol HCl, GENZ-123346) showed different antiviral effects on the SARS-CoV-2-infected Calu3 cells (Fig. 5). Remdesivir showed the highest effect, Ribavirin and GENZ-123346 showed similar effects, and Ambroxol HCl was the least effective drug, but still showed ~ 0.5 log reduction in viral replication.

**Fig. 6:**
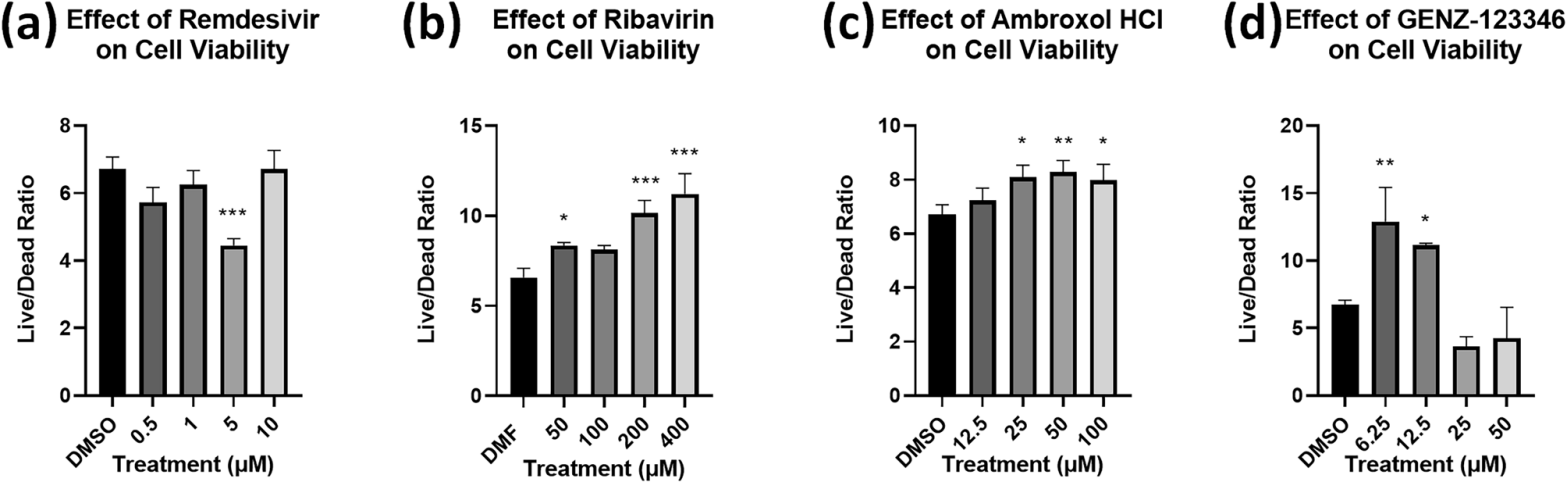
Concentration-dependent effects of drug compounds on host cell viability (live/dead ratio) for **a** Remdesivir, **b** Ribavirin, **c** Ambroxol HCl, and **d** GENZ-123346. Remdesivir showed no notable changes to cell viability, while both Ribavirin and GENZ-123346 showed cytoprotective effects with increased viability. GENZ-123346 showed increased viability at low concentration and vice versa at high concentration (One way ANOVA with Dunnett’s multiple comparisons test: * = p ≤ 0.05, ** = p ≤ 0.01, *** = p ≤ 0.001)

In addition, we investigated the cytotoxic effects of the drug compounds on healthy Calu3 cells. The live/dead ratio results are shown in Fig. 6, and the normalized live and dead cell counts are shown in Supplementary Fig. 3. Remdesivir seemed to have no effects on the live/dead ratio except for 5 μM. Both Ambroxol HCl and Ribavirin showed cytoprotective effects as the concentration increased. Finally, GENZ-123346 showed cytoprotective effects at low concentrations, but significant cytotoxic effects at high concentrations.

## Discussion

**Fig. 7:**
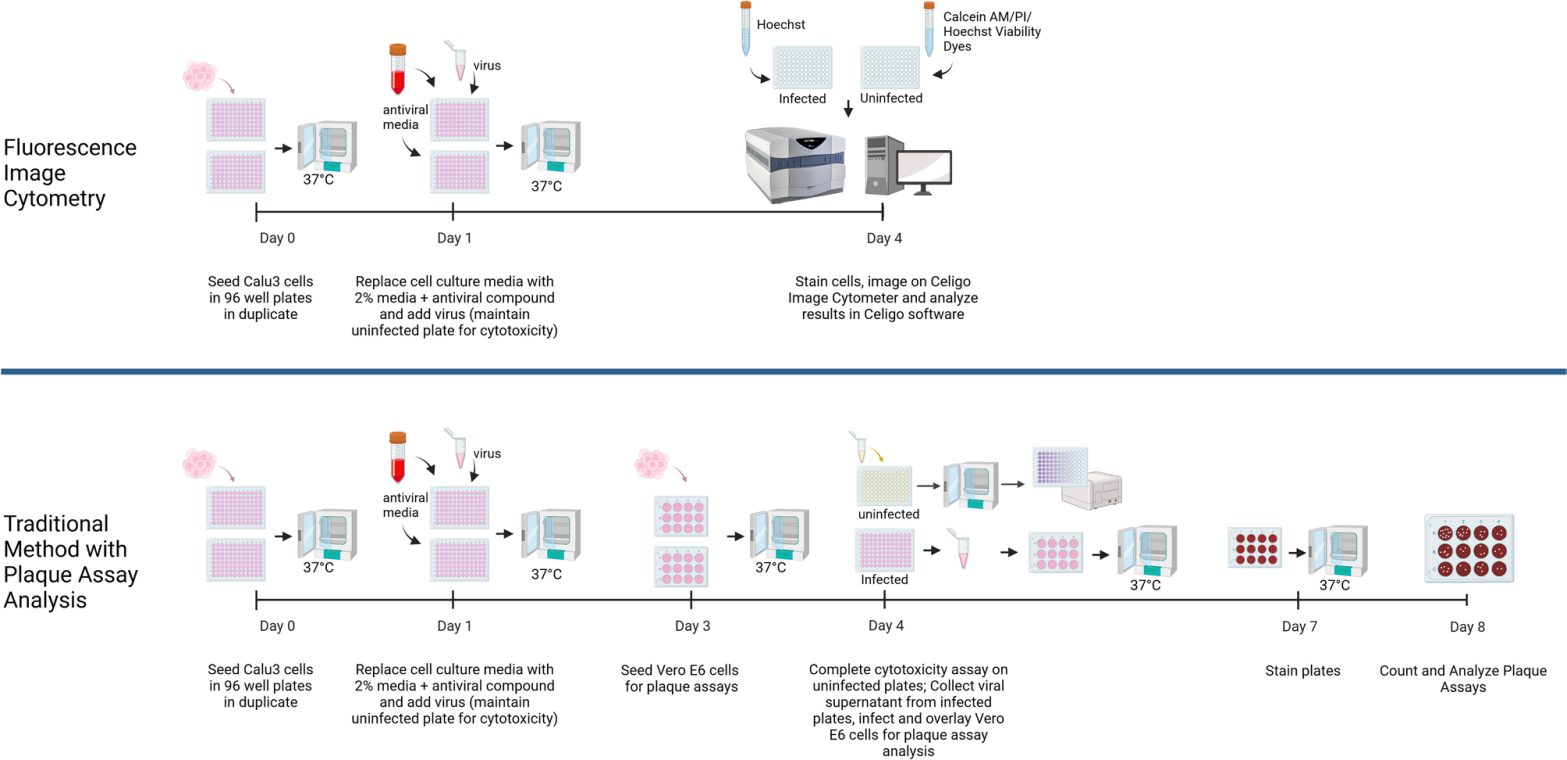
Side-by-side comparison of time-to-completion of the cytometry-based method of antiviral testing versus traditional assays. Compared to traditional methods using plaque assay analysis to evaluate antiviral efficacy of compounds, the fluorescence cytometry methodology outlined in this paper shortens processing time by an average of 3–4 days and reduces the number of overall steps for the user while providing similar data output and digital image storage. Image generated in Biorender

The SARS-CoV-2 viral outbreak has spread rapidly across the globe prompting the urgent need for discovery of novel antiviral and vaccine candidates. To increase the efficiency of screening of viable candidates, it is important to develop a high-throughput antiviral testing method that can show significant improvement from the traditional assays such as PRNT, plaque assay, or RT-PCR. Plate-based image cytometry can increase the speed of data acquisition and analysis in a high-speed and high-throughput manner, enables bright field- and fluorescence-based detection, as well as provide image-based digital records (Fig. 7).

We demonstrated the use of the Celigo Image Cytometer for high-throughput SARS-CoV-2 antiviral testing by first optimizing the methodology by selecting a host cell line and optimizing the MOI and seeding density. Of the five host cell lines tested, only Calu3 showed a high increase in normalized mNeonGreen positive cells on an order of ~ 2 logs without inducing cytopathic effects on the host cells. The ACE2-A549 showed greater than 2 decades of increase in mNeonGreen positive cells, however, severe cytopathic effects and cell death were observed rendering this cell line inappropriate for this assay. The MOI of 0.1 for Calu3 showed the most stable infection rate, thus was selected for the testing method. Due to variations of cell counts during initial seeding and potential cell loss after infection, the total cell counts were used to normalize the mNeonGreen positive cell counts to minimize the variations in the results.

The seeding density required optimization to ensure the host cells are not over-confluent 96 h post-seeding, which can stress the cells and potentially affect the infectivity of SARS-CoV-2 virus on the host cells. Seeding densities at 4 × 10^4^ cells/well or above showed confluence percentages approximately 85 – 98%, which was too high for the assay. Both 1 × 10^4^ and 2 × 10^4^ seemed to be in the appropriate range for cell seeding. Interestingly, the seeded Calu3 cells did not proliferate, and remained consistent from 24 – 96 h, which indicated that Calu3 is a slow growing cell type and is consistent with other published studies [30].

After optimizing the high-throughput antiviral testing method, we examined the cytotoxicity and antiviral activity of four pre-selected compounds repurposed for COVID-19 treatment. Both Remdesivir and Ribavirin have previously shown in vitro and had clinical efficacy against the SARS-CoV-2 [25]. In this experiment, those results were recapitulated using the image cytometry method showing strong concentration-dependent antiviral effects against SARS-CoV-2 at higher concentrations. With the exception of the 5 µM treatment of Remdesivir, all concentrations tested of Remdesivir, and Ribavirin had no cytotoxic effects on Calu3 cells. Ambroxol HCl and GENZ-123346 also showed antiviral effects at the two highest concentrations, however they differed in their effects on cell viability. Ambroxol HCl treatment improved cell viability, suggesting it may have a cytoprotective effect, whereas GENZ-123346 was cytoprotective at low concentrations, and cytotoxic at concentrations at 25 µM and above. Due to its ability to inhibit acid sphingomyelinase activity, Ambroxol HCl was previously shown to prevent entry and viral replication of a vesicular stomatitis virus SARS-CoV-2 pseudovirus into Vero E6 cells and Caco-2 cells at concentrations of 25 µM [31], however, our results showed only modest inhibition of viral replication at 50 µM and 100 µM using full-length SARS-CoV-2 virus in Calu3 cells. This suggests that while use of pseudovirus like pp-VSV-SARS-CoV-2 containing the SARS-CoV-2 spike protein allows for BSL-2 level investigations of SARS-CoV-2 mechanisms of entry, additional follow up of antiviral agents using full-length virus in BSL-3 conditions is necessary. Additionally, our results also may suggest that the IC_50_ of Ambroxol HCl against acid sphingomyelinase is cell-line dependent, and higher concentrations may be needed to prevent viral entry in lung epithelial cells (Calu3) compared to colon epithelial cells (Caco-2), an intriguing finding that has yet to be investigated. GENZ-123346 was previously shown to effectively inhibit SARS-CoV-2 replication at concentrations of 10 µM and above [32], however, in our experiments, it was only modestly effective at reducing viral replication at 12.5 µM, and showed cytotoxicity starting at concentrations above 25 µM, with complete cell death at the 50 µM concentration. These results were striking considering previously reported cell viability at concentrations as high as 400 µM [33], and warrant additional testing and scrutiny of GENZ-123346 prior to its consideration as a potential antiviral candidate against SARS-CoV-2 as previously suggested (32).

 In conclusion, the development of an image-based high-throughput antiviral testing method allowed rapid characterization of potential antiviral drug candidates, which enabled direct quantification of the antiviral effects on SARS-CoV-2 viral infectivity and their cytotoxic and/or cytoprotective effects to the host cells, which are both critical to ensure the efficacy and safety of the potential antiviral drug candidates. Furthermore, the versatility of the methodology developed in this work may also be adopted for screening antiviral candidates for other diseases. As examples, the plate-based image cytometry methods outlined here can be adapted to stain for viral proteins instead of using a reporter virus for quantifying replication of wild type virus or variants of concern, or the cytotoxicity assays described can be modified and utilized to calculate viral titer (TCID_50_/mL). Additionally, the methodology described herein can be further adapted for use in studies examining the effects of antiviral compounds on viral replication kinetics and viral spread as this live-cell imaging approach allows for the study of the same cell populations over multiple timepoints and days.

### Supplementary Information

Below is the link to the electronic supplementary material.

Supplementary file1 Supplementary Fig. 1. Bright field and fluorescent overlay images of mNeonGreen SARS-CoV-2-infected Fadu and Detroit 562 at MOIs 0.001, 0.01, 0.1, and 1. Visually, both cell lines showed low to no permissibility to the SARS-CoV-2 virus. (TIF 3136 KB)

Supplementary file2 Supplementary Fig. 2. The bright field images of Calu3 confluency at seeding densities 6 × 104, 8 × 104, and 1 × 105 cells/well from 24 to 96 h. Visually, these densities are over-confluent. (TIF 2741 KB)

Supplementary file3 Supplementary Fig. 3. Concentration-dependent effects of drug compounds on live and dead cell count for (a, e) Remdesivir, (b, f) Ribavirin, (c, g) Ambroxol HCl, and (d, h) GENZ-123346 (One way ANOVA with Dunnett’s multiple comparisons test: * = p ≤ 0.05, ** = p ≤ 0.01, *** = p ≤ 0.001, **** = p ≤ 0.0001). (TIF 600 KB)

## Data Availability

Supplementary material related to this article can be found in the online version.
