# Supplementary figures and images for: High-Throughput SARS-CoV-2 Antiviral Testing Method Using the Celigo Image Cytometer
Source: J Fluoresc. 2023 Jun 13;34(2):561–70. doi: 10.1007/s10895-023-03289-x (PMC10261830; doi:10.1007/s10895-023-03289-x)

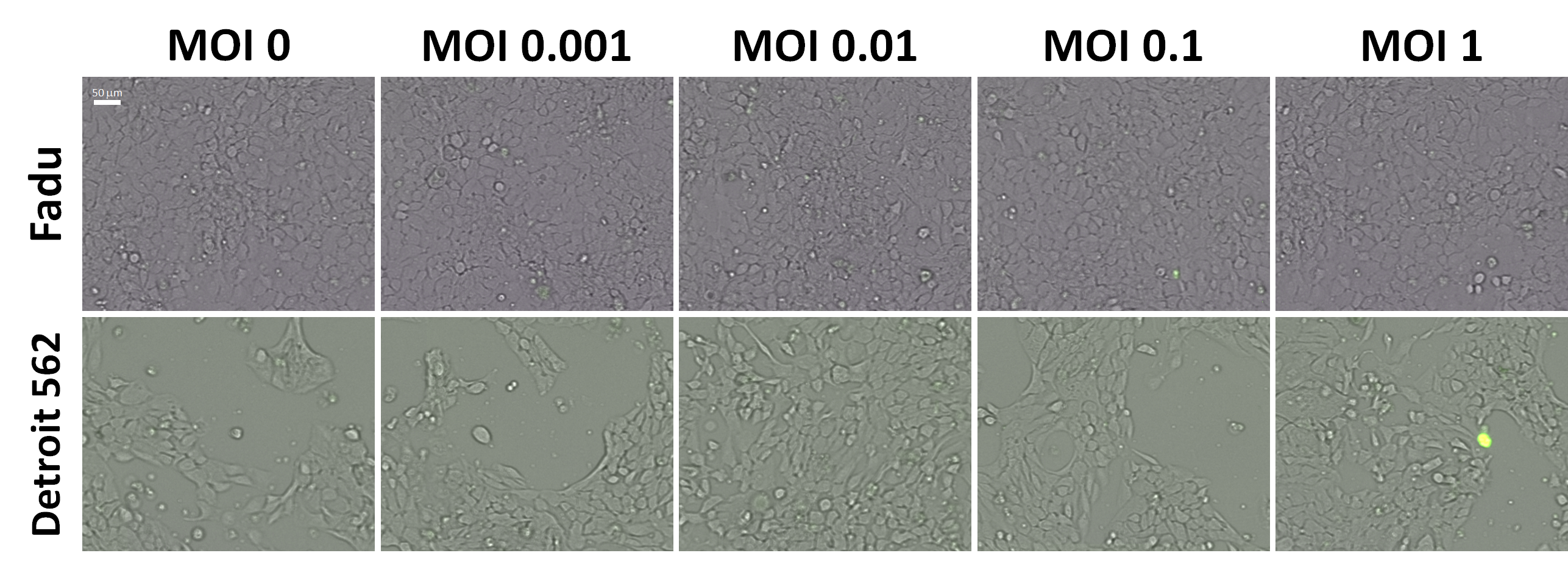

Supplement: Supplementary file 1 — Supplementary file1 Supplementary Fig. 1. Bright field and fluorescent overlay images of mNeonGreen SARS-CoV-2-infected Fadu and Detroit 562 at MOIs 0.001, 0.01, 0.1, and 1. Visually, both cell lines showed low to no permissibility to the SARS-CoV-2 virus. (TIF 3136 KB) [file 10895_2023_3289_MOESM1_ESM.tif]

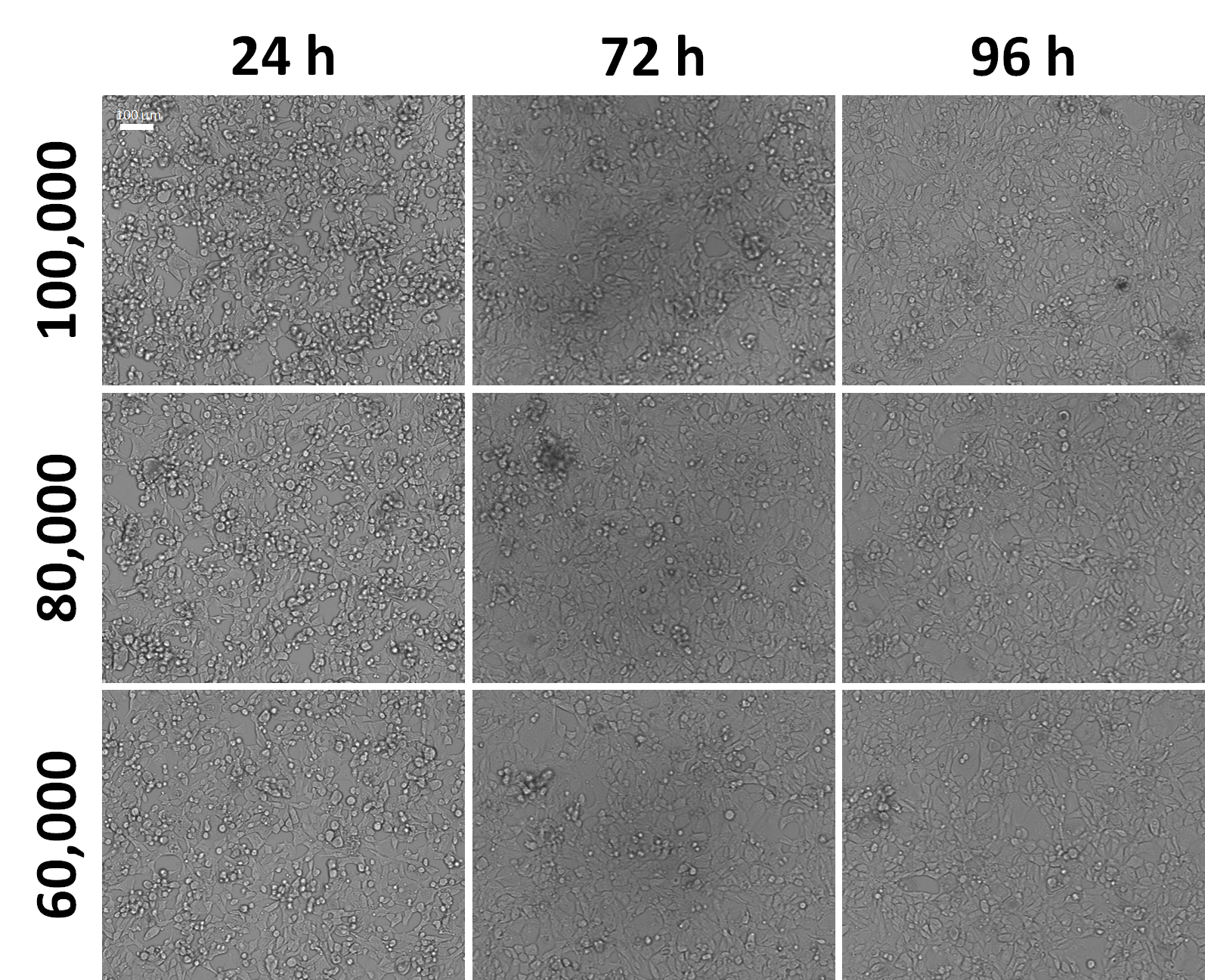

Supplement: Supplementary file 2 — Supplementary file2 Supplementary Fig. 2. The bright field images of Calu3 confluency at seeding densities 6 × 10^4^, 8 × 10^4^, and 1 × 10^5^ cells/well from 24 to 96 h. Visually, these densities are over-confluent. (TIF 2741 KB) [file 10895_2023_3289_MOESM2_ESM.tif]

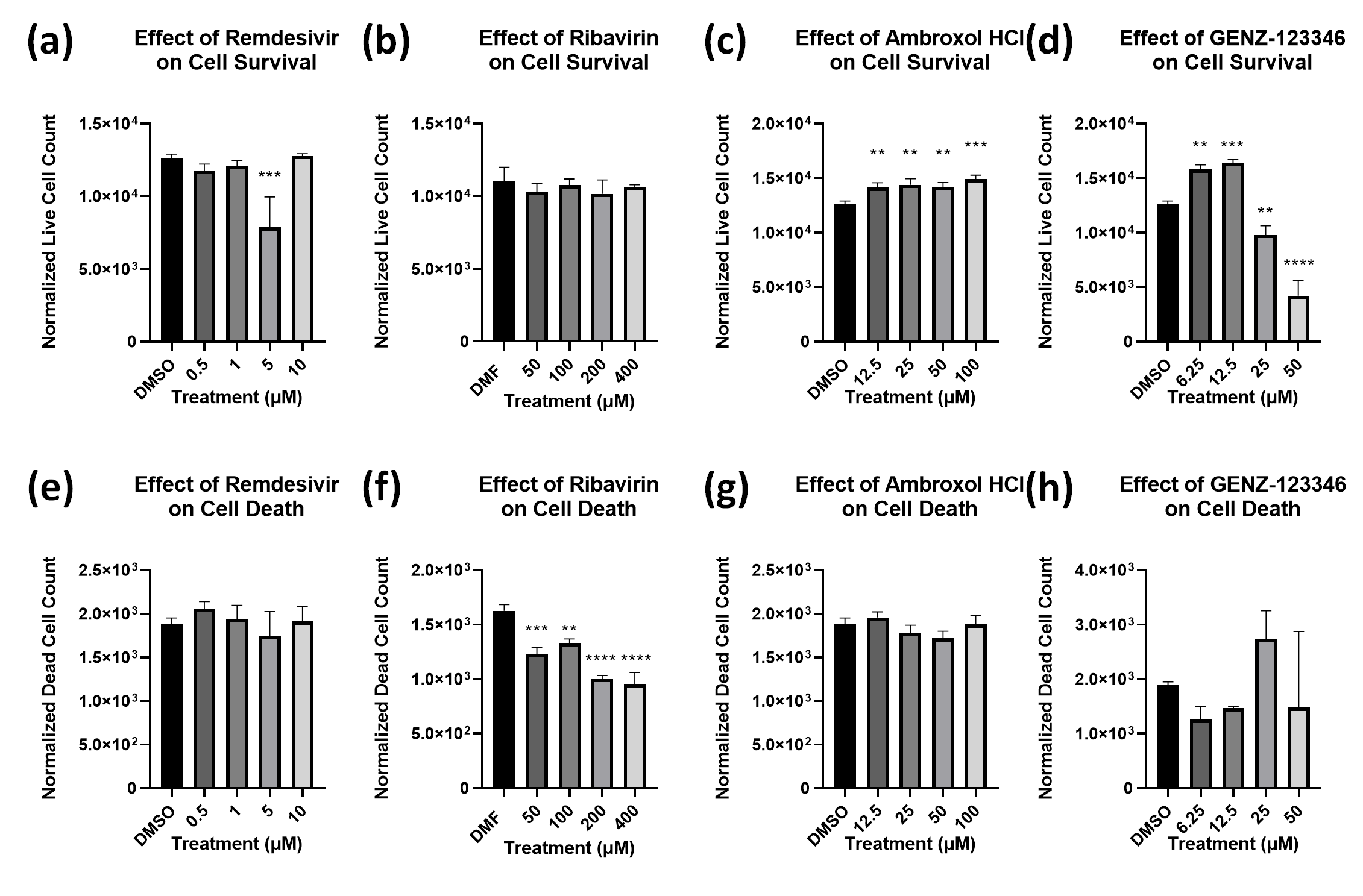

Supplement: Supplementary file 3 — Supplementary file3 Supplementary Fig. 3. Concentration-dependent effects of drug compounds on live and dead cell count for (a, e) Remdesivir, (b, f) Ribavirin, (c, g) Ambroxol HCl, and (d, h) GENZ-123346 (One way ANOVA with Dunnett’s multiple comparisons test: * = p ≤ 0.05, ** = p ≤ 0.01, *** = p ≤ 0.001, **** = p ≤ 0.0001). (TIF 600 KB) [file 10895_2023_3289_MOESM3_ESM.tif]
